# Social network analysis of nationwide interhospital emergency department transfers in Taiwan

**DOI:** 10.1038/s41598-023-29554-4

**Published:** 2023-02-09

**Authors:** Chu-Lin Tsai, Ming-Tai Cheng, Shu-Hsien Hsu, Tsung-Chien Lu, Chien-Hua Huang, Yueh-Ping Liu, Chung-Liang Shih, Cheng-Chung Fang

**Affiliations:** 1 Department of Emergency Medicine, National Taiwan University Hospital, 7 Zhongshan S. Rd, Taipei, 100 Taiwan; 2 Department of Emergency Medicine, College of Medicine, National Taiwan University, Taipei, Taiwan; 3 Ministry of Health and Welfare, Taipei, Taiwan

**Keywords:** Health care, Health services, Public health, Statistical methods

## Abstract

Transferring patients between emergency departments (EDs) is a complex but important issue in emergency care regionalization. Social network analysis (SNA) is well-suited to characterize the ED transfer pattern. We aimed to unravel the underlying transfer network structure and to identify key network metrics for monitoring network functions. This was a retrospective cohort study using the National Electronic Referral System (NERS) database in Taiwan. All interhospital ED transfers from 2014 to 2016 were included and transfer characteristics were retrieved. Descriptive statistics and social network analysis were used to analyze the data. There were a total of 218,760 ED transfers during the 3-year study period. In the network analysis, there were a total of 199 EDs with 9516 transfer ties between EDs. The network demonstrated a multiple hub-and-spoke, regionalized pattern, with low global density (0.24), moderate centralization (0.57), and moderately high clustering of EDs (0.63). At the ED level, most transfers were one-way, with low reciprocity (0.21). Sending hospitals had a median of 5 transfer-out partners [interquartile range (IQR) 3–7), while receiving hospitals a median of 2 (IQR 1–6) transfer-in partners. A total of 16 receiving hospitals, all of which were designated base or co-base hospitals, had 15 or more transfer-in partners. Social network analysis of transfer patterns between hospitals confirmed that the network structure largely aligned with the planned regionalized transfer network in Taiwan. Understanding the network metrics helps track the structure and process aspects of regionalized care.

## Introduction

Transferring patients between emergency departments (EDs) is a complex and understudied issue. A myriad of factors are involved, including the capability of the sending hospital, the capacity of the receiving hospital, risks of interhospital transfer, and perhaps most importantly, patient preferences and outcomes^1^. Once the transfer decision is made, the choice of destination hospital often depends on a formal or informal regional transfer network^2,3^. Regionalization of care (i.e., sending patients to specialized centers with appropriate resources) has been shown to result in better patient outcomes for time-sensitive conditions, such as major trauma, ST-segment elevation myocardial infarction (STEMI), and stroke^2,4–9^. However, how the network functions is often unknown, and quantitative network metrics are lacking.

Social network analysis (SNA) is an emerging tool in social science and medicine that can characterize the connections between patients, healthcare providers, hospitals within a healthcare system^10–12^. Not only can this technique visualize the existence of a network, but it also provides metrics that quantify the strengths of the relationships of members in a network^13,14^. For example, SNA has been used to study the spread of obesity in a community^15^, news-sharing behaviors about coronavirus on Twitter^16^, inpatient services flow in a hospital^17^, intensive care unit (ICU) to ICU transfers^18^, and patient-sharing networks of physicians^10^. Recent reviews have begun to cover the basics of SNA in health care and health services research^19,20^. To the best of our knowledge, there have been no studies employing SNA to approach interhospital ED transfers, a complex system problem that is amenable to such a technique.

In the current study, we analyzed nationwide ED transfer data with the goals to (1) characterize the transfer pattern and the underlying network structure; and to (2) identify key network metrics to understand how the network functions. A deeper understanding of the transfer network would allow clinicians, researchers, and policymakers to identify unusual ED transfer patterns and investigate subsequent patient outcomes for quality improvement purposes.

## Methods

### Study design and setting

**Figure 1:**
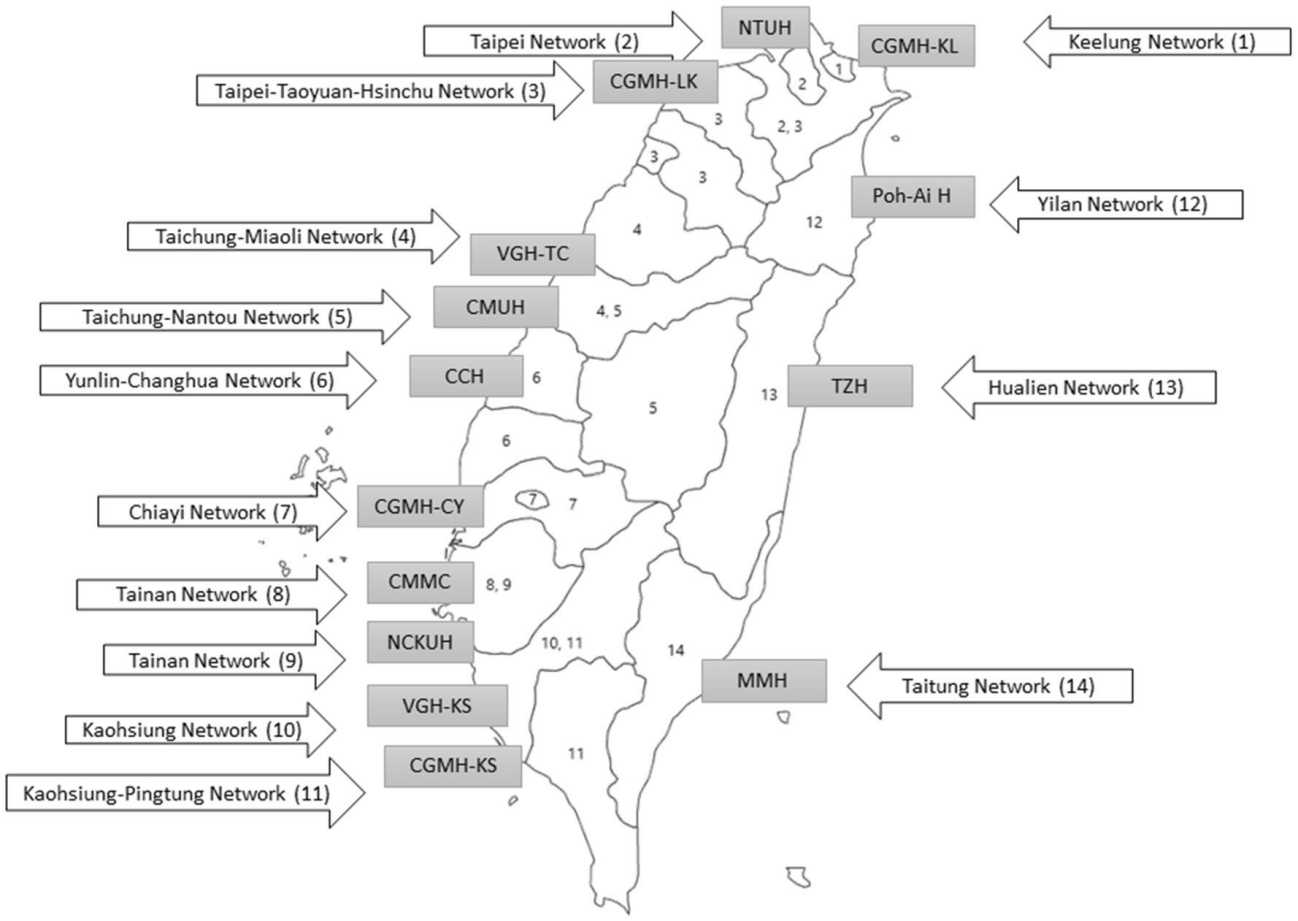
The 14 emergency care referral networks designated by the Ministry of Health and Welfare of Taiwan. Each of the 14 referral networks has a base hospital, which is highlighted in gray. The figure was created using Microsoft PowerPoint 2019 (www.microsoft.com).

Taiwan’s national health insurance (NHI) is a single-payer system that provides universal, mandatory coverage for 23 million people on the island. The system is administered by the Ministry of Health and Welfare (MOHW), which sets policy, determines payroll-based premiums (subject to approval by Congress), and pays contracted providers and hospitals^21^. For this study, we conducted a retrospective cohort study using data from the National Electronic Referral System (NERS) in Taiwan. The NERS is an online, national electronic transfer platform founded by the MOHW in 2012 in response to several controversies surrounding patient dumping and inappropriate transfers. Among them was an incident where a severely beaten four-year-old girl was bounced from hospital to hospital and was declared brain-dead in a hospital 120 + miles away^22^. The online platform is used to coordinate and document ED transfers in the country (Online Supplementary eFigure 1). Since then, the MOHW has designated 14 emergency care referral networks (Fig. 1) that initially included 193 hospital-based EDs across the nation as the first step toward regionalization of emergency care^23^. The EDs nationwide are accredited by the Joint Commission of Taiwan periodically and are categorized as basic, intermediate, and advanced EDs by their capabilities and resources available across all conditions^24^. The capability to manage certain time-sensitive conditions (e.g., major trauma, STEMI, stroke) is also designated to supplement the general categorization. Each of the 14 referral networks has a base hospital with or without co-base hospital(s). Both base and co-base hospitals are advanced EDs that serve as a hub for receiving transfers. All advanced EDs have the capability to manage major trauma, STEMI, and stroke. The capacity of the base hospitals and the size of the population served are listed in the Online Supplementary eTable 1. The Central Mountain Range separates Taiwan into eastern and western parts. In general, the western side of Taiwan is more populous and developed than its eastern side (Yilan, Hualien, and Taitung networks). Thus, more medical resources are available on the west side of the island.

Hospital abbreviations in the order of the network number: CGMH-KL = Chang Gung Memorial Hospital-Keelung; NTUH = National Taiwan University Hospital; CGMH-LK = Chang Gung Memorial Hospital-Linkou; VGH-TC = Veterans General Hospital-Taichung; CMUH = China Medical University Hospital; CCH = Changhua Christian Hospital; CGMH-CY = Chang Gung Memorial Hospital-Chiayi; CMMC = Chi-Mei Medical Center; NCKUH = National Cheng-Kung University Hospital; VGH-KS = Veterans General Hospital-Kaohsiung, CGMH-KS = Chang Gung Memorial Hospital-Kaohsiung, TZH = Tzu-Chi Hospital; MMH = MacKay Memorial Hospital.

For each ED transfer, the sending hospital must enter structured information on the NERS online platform, including patient demographics, up to 3 diagnoses, transfer reasons, transfer time, and vital signs upon ED departure. Similarly, the receiving hospital must enter information on diagnosis, arrival time, vital signs on ED arrival, and patient disposition. The NERS data are maintained by the Taiwan Society of Emergency Medicine (TSEM). For this study, we requested de-identified data from the TSEM and received an exemption from our institutional review board. All methods were performed in accordance with the relevant guidelines and regulations, including the Declaration of Helsinki.

### Study population

All interhospital ED transfers from January 1, 2014, to December 31, 2016, were included, totaling 218,760 ED transfers during the 3-year study period. The contemporaneous number of total ED visits was 22,033,309^25^; thus, the average transfer rate was about 1% per year.

### Measurements

Transfer characteristics were extracted, including patient demographics, clinical conditions, transfer and receiving dates/times, transfer reasons, sending and receiving hospitals, and final patient disposition. Data underwent rigorous electronic cleaning and invalid data were set to missing values. The clinical conditions were coded as International Classification of Diseases, Ninth Revision, Clinical Modification [ICD-9-CM], or ICD-10 codes, as appropriate. The primary diagnosis was grouped into clinically meaningful categories using the Clinical Classification Software (CCS)^26^. Each sending and receiving hospital has a national hospital number that can be readily used for SNA. We also obtained hospitals’ locations, including addresses, longitudes, and latitudes, from the MOHW’s annual hospital directory and Google Maps searches.

### Terminology of network analysis

#### Node-level statistics

For SNA in our study, each hospital was a node, and each transfer represented a line/connection (or tie) between hospitals. Connections were directional, and the direction represented transferring patients from the sending hospital to the receiving hospital. A dyad is a pair of nodes; dyads are asymmetrical when there is a connection from one node to the other but not the other way around. Connections were valued in our study, with the value representing the number of transfers (i.e., strengths of transfer relationships). The degree of a node is the number of ties a node has. For a directed network, in-degree is the number of ties that a node receives from others, and out-degree is the number of ties that a node sends to others. In the context of this study, these two terms are synonymous with “transfer-in partners” and “transfer-out partners,” respectively. The betweenness centrality of a node is the number of shortest paths among all other nodes that pass through this node. In this directed network analysis, a standardized betweenness centrality was computed by dividing betweenness by (N-1)(N-2). The farness of a node is the sum of its distances to all other nodes. Closeness is then defined as the inverse of the farness. In this study, a standardized closeness centrality was computed by multiplying the closeness by (N-1). The local clustering coefficient of a node is the proportion of ties between the nodes within its neighborhood divided by the maximally possible number of ties between them.

#### Network-level statistics

Network-level statistics were computed, including global density, in-degree centralization, clustering, and reciprocity. All of the network-level metrics ranged from 0 to 1, with 1 being the maximum value. The global density is the fraction of ties in a network relative to the maximum possible number of ties. The in-degree centralization measures how centralized a network is. For example, if all nodes are connected through only one node (single central hub), the in-degree centrality would be 1. A network clustering coefficient is a measure of the extent to which nodes in a network tend to cluster together. In this study, an average clustering coefficient was computed by taking the average of all local clustering coefficients for the nodes in the network. Reciprocity is a specific quantity for a directed network that measures the tendency of pairs of nodes to form mutual connections between each other.

#### Sociogram

Sociograms were constructed by connecting the nodes with lines to visualize the relationships between hospitals better. We depicted two types of sociograms: the overall sociogram that included all 199 EDs with a minimum number of 1 transfer, and the skeleton sociogram that included 192 EDs with a minimum of 36 transfers over three years (≥ 1 transfer per month). The skeleton sociogram was further divided into one with medium ties (36–365 transfers over three years) and the other with strong ties (> 365 transfers over three years). To better understand the changes in the network over time, we also depicted the overall sociogram per year. A video clip was made to demonstrate the changes in the network throughout the study period.

### Outcome measures

The key network statistics included, at the ED level, the number of nodes (EDs), the number of ties (connections between EDs), in-degree, out-degree, clustering, betweenness, and closeness. At the network level, the statistics included global density, in-degree centralization, clustering, and reciprocity.

### Statistical analysis

Summary statistics are presented as proportions (with 95% confidence intervals [CI]), means (with standard deviations [SD]), or medians (with interquartile ranges [IQR]). Social network analysis was used to characterize the underlying transfer network structure and to compute network statistics. Sociograms, incorporating each hospital’s geographic coordinate information, were plotted for mapping purposes. All analyses were performed using Stata 16.0 software (StataCorp, College Station, TX) with the nwcommands package.

## Results

There were a total of 218,760 ED transfers during the 3-year study period. The characteristics of the transfers are shown in Table 1. The mean age was 56 years (range 0–111), and 39% were female. The vast majority of the transfers occurred in the daytime or evening, 29% over the weekends, and 27% during the winter months. The most common reasons for transfer were requests from patients or families, followed by a lack of on-call specialist coverage and capacity issues at the sending hospital. The most commonly transferred conditions included acute stroke, pneumonia, traumatic brain injury, fractures, and myocardial infarction. Nine percent of the transfers underwent surgery at the receiving hospital, and most of the transfers were hospitalized. Of note, about 6% of the transferred patients were discharged with outpatient follow-up at the receiving hospital.

**Table 1:** Characteristics of emergency department transfers.

Variable	N = 218,760
Age, mean (SD), year	55.7 (23.8)
Female sex, n (%)	86,113 (39.4)
Transfer out time, n (%)	
7 a.m. to 3 p.m.	87,253 (39.9)
3 p.m. to 11 p.m.	90,765 (41.5)
11 p.m. to 7 a.m.	40,742 (18.6)
Transfer out during the weekend, n (%)	62,927 (28.8)
Transfer-out season, n (%)	
Spring (Mar. to May)	53,177 (24.3)
Summer (Jun. to Aug.)	54,476 (24.9)
Fall (Sep. to Nov.)	52,748 (24.1)
Winter (Dec. to Feb.)	58,359 (26.7)
Transfer reason, n (%)*	
Request from patients or families	91,909 (43.0)
Specialist not available	71,016 (33.2)
Hospital capability problem	49,797 (23.3)
Other	1152 (0.5)
Clinical condition, n (%)	
Acute cerebrovascular disease	18,638 (8.5)
Pneumonia	10,500 (4.8)
Intracranial injury	9079 (4.2)
Fracture of upper limb	8488 (3.9)
Fracture of lower limb	8357 (3.8)
Acute myocardial infarction	7613 (3.5)
Surgery at receiving hospital, n (%)	20,446 (9.4)
Disposition at receiving hospital, n (%)	
Admission to ward	92,265 (42.2)
Observation and ongoing ED management	64,292 (29.4)
ICU admission	48,980 (22.4)
Discharged with outpatient follow-up	12,051 (5.5)
Transfer out again	1053 (0.5)
Transfer back	119 (0.1)

*SD* standard deviation, *ED* emergency department, *ICU* intensive care unit.

*Available for 213,874 transfers.

**Figure 2:**
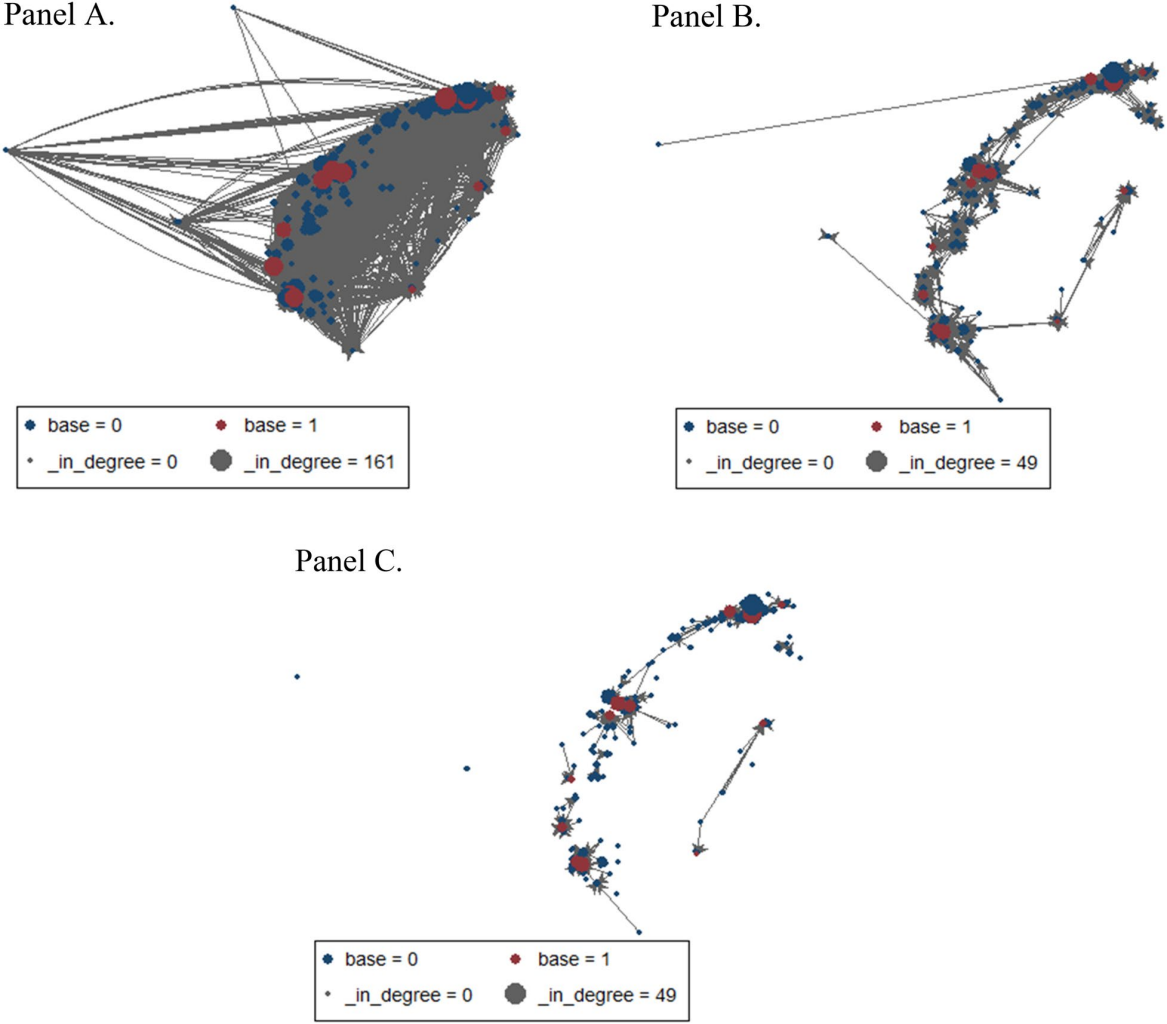
Sociograms illustrating interhospital emergency department transfers. The size of a node is proportional to its in-degree. Red circles indicate base hospitals, and blue circles indicate non-base hospitals. (**Panel A**) The overall sociogram. The red circles indicate the 14 base hospitals. (**Panel B**) The skeleton sociogram, medium ties, with 36 to 365 transfers over three years (approximately 1–10 transfers per month). (**Panel C**) The skeleton sociogram, strong ties, with > 365 transfers over three years (> 1 transfer every three days). The figures were created using Stata 16.0 (stata.com).

In the overall sociogram, there were a total of 199 EDs with 9,516 transfer ties between EDs. The 9,516 connections essentially depicted the shape of the main island of Taiwan and three offshore islands (Fig. 2, panel A). The 14 base hospitals were intended to serve as the transfer hub in each regional transfer network. The regionalized networks gradually emerged in the skeleton view (Fig. 2, panels B,C) as weak ties were removed. In Panel C, the nodes with the strongest ties (> 365 transfers over three years) were highly regionalized and locally clustered. The entire network demonstrated a multiple hub-and-spoke, regionalized pattern, which was mostly consistent with the government-planned regionalized transfer network (Fig. 1). The in-degrees of the base hospitals on the western side were larger than those on the eastern side, as reflected by the larger sizes of the nodes. A linear regression confirmed a positive relationship between regional population size and in-degree (β =  + 5.5 in-degrees per million residents, *p* < 0.001).

**Figure 3:**
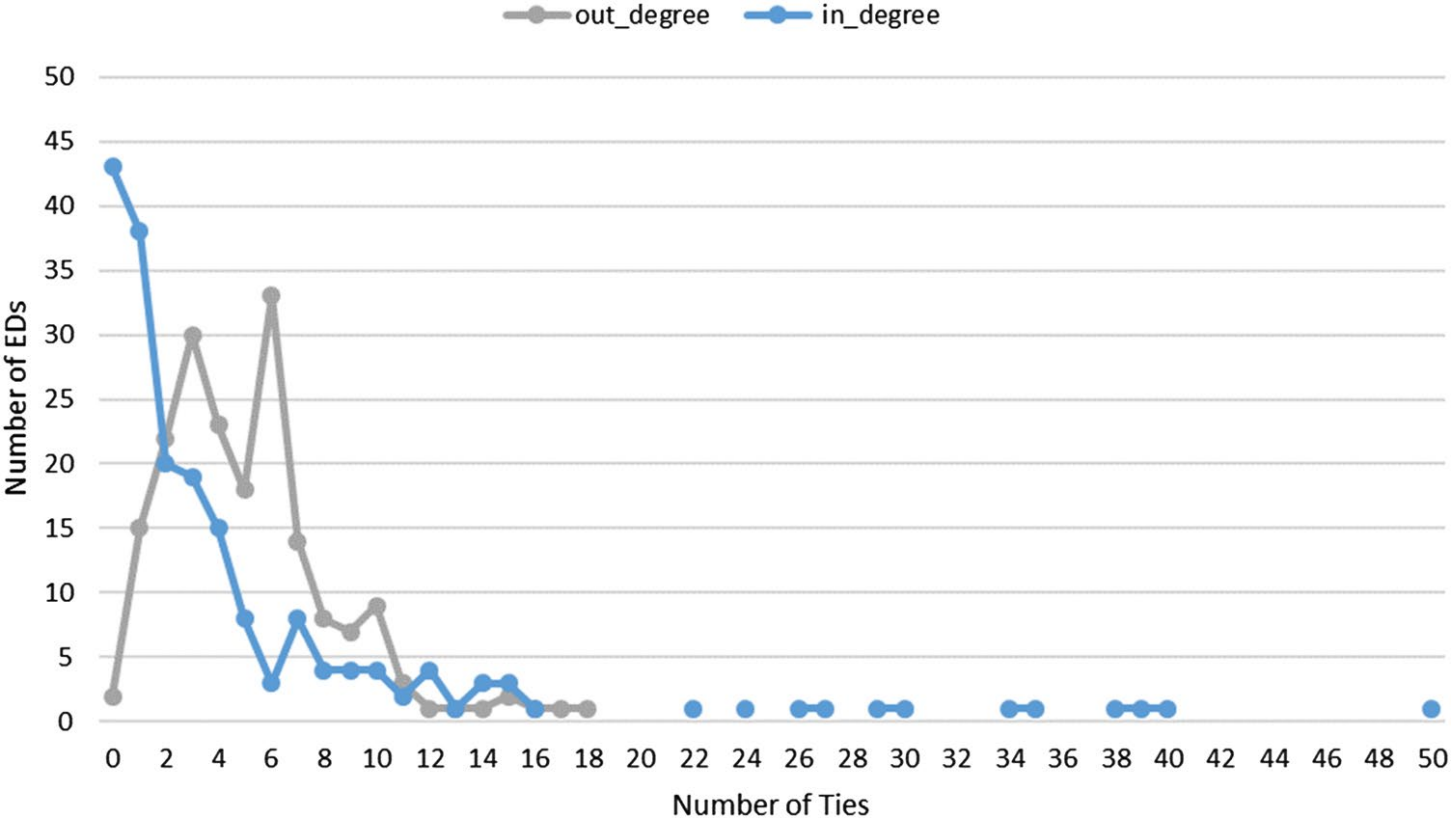
The distribution of in-degrees (transfer-in partners) and out-degrees (transfer-out partners) in the skeleton network. Few hospitals (mostly base hospitals in the long tail) received patients from a disproportionally high number of partners.

The SNA of the overall network (Table 2) showed low global density (0.24), moderate in-degree centralization (0.57), and moderately high average clustering (0.63). Among the EDs with medium to strong ties (i.e., skeleton network), most ties were asymmetric dyads, with low reciprocity (0.21). The distribution of in-degrees (transfer-in partners) and out-degrees (transfer-out partners) in the skeleton network is shown in Fig. 3. Sending hospitals had a median of 5 (IQR 3–7) transfer-out partners across all conditions, while receiving hospitals a median of 2 (IQR 1–6) transfer-in partners. Few hospitals received patients from a disproportionally high number of partners, suggesting a scale-free network pattern. For example, a total of 16 receiving hospitals, all of which were designated base or co-base hospitals, had 15 or more transfer-in partners.

**Table 2:** Network-level statistics of the overall and skeleton networks.

	Overall network	Skeleton network (≥ 1 transfer per month)
Nodes (number of hospital-based EDs)	199	192
Ties (connections between hospital-based EDs)	9516	997
Density	0.24	0.03
In-degree centralization	0.57	0.24
Average clustering coefficient	0.63	0.59
Asymmetric/mutual dyads	4154/2681	651/173
Reciprocity	0.39	0.21
Standardized betweenness centrality, mean (SD)	0.004 (0.007)	0.007 (0.025)
Standardized closeness centrality, mean SD	0.61 (0.08)	0.28 (0.04)

**Figure 4:**
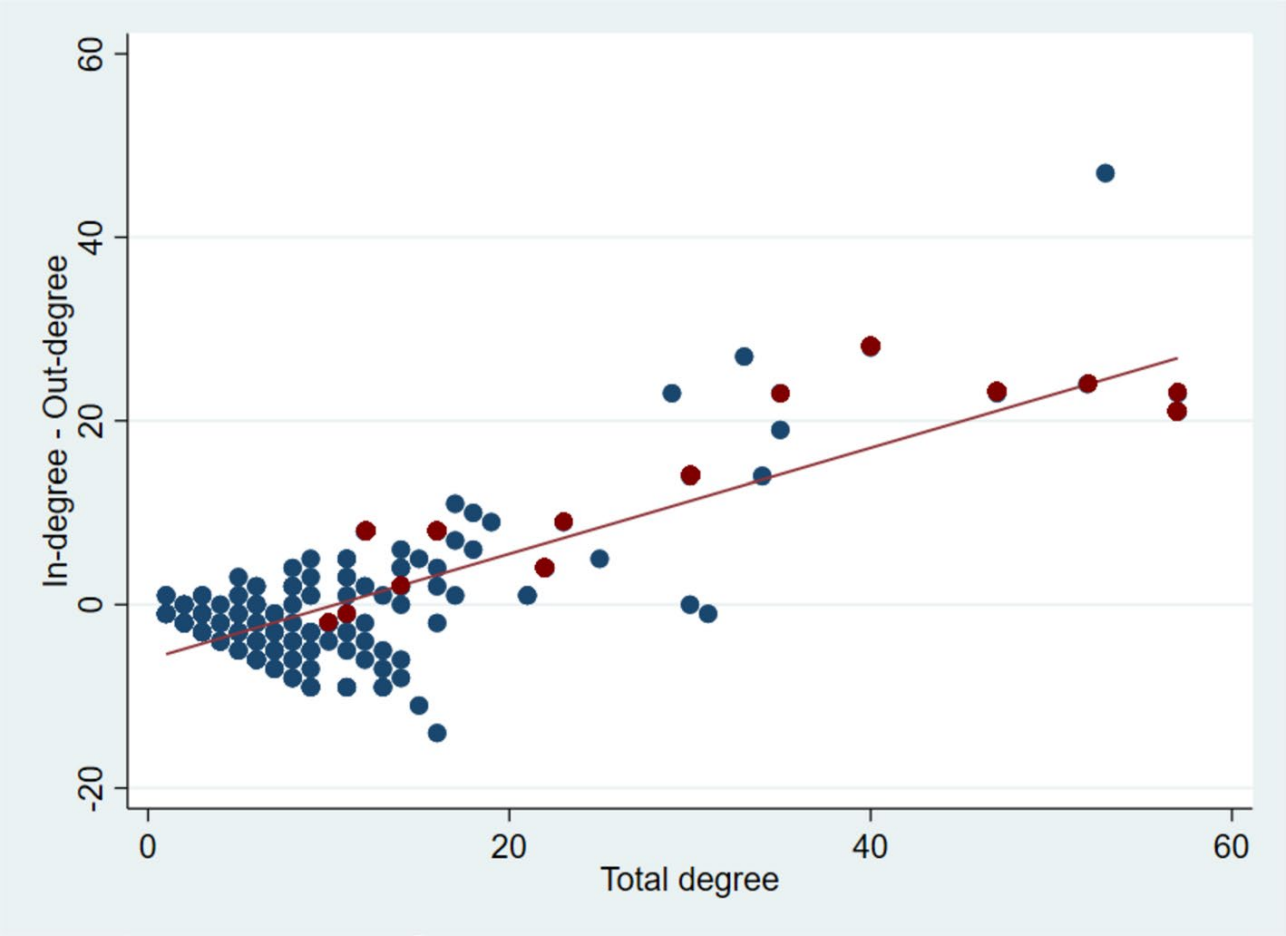
The net connectivity of nodes. Most base (“receiver”) hospitals (in red) have positive traffic (in-degree > out-degree), whereas some “distributor” hospitals have negative traffic. A total degree is the sum of in-degree and out-degree.

Figure 4 shows the in-degree and out-degree balance, i.e., the net connectivity of nodes. Similarly, most of the base hospitals were “receivers” (in-degree > out-degree), as opposed to “distributors” (out-degree > in-degree). For the top 5% distributor EDs, the patients they transferred out had a small but significantly higher percentage of being directly discharged from the receiving ED (6.2% vs. 5.4%, *p* < 0.001). In terms of the distributions of other node-level statistics (Online Supplementary Figures 2–4), the distribution of betweenness also showed a power-law distribution, with base hospitals in the long tail. By contrast, the distribution of closeness and clustering did not follow this pattern.

**Figure 5:**
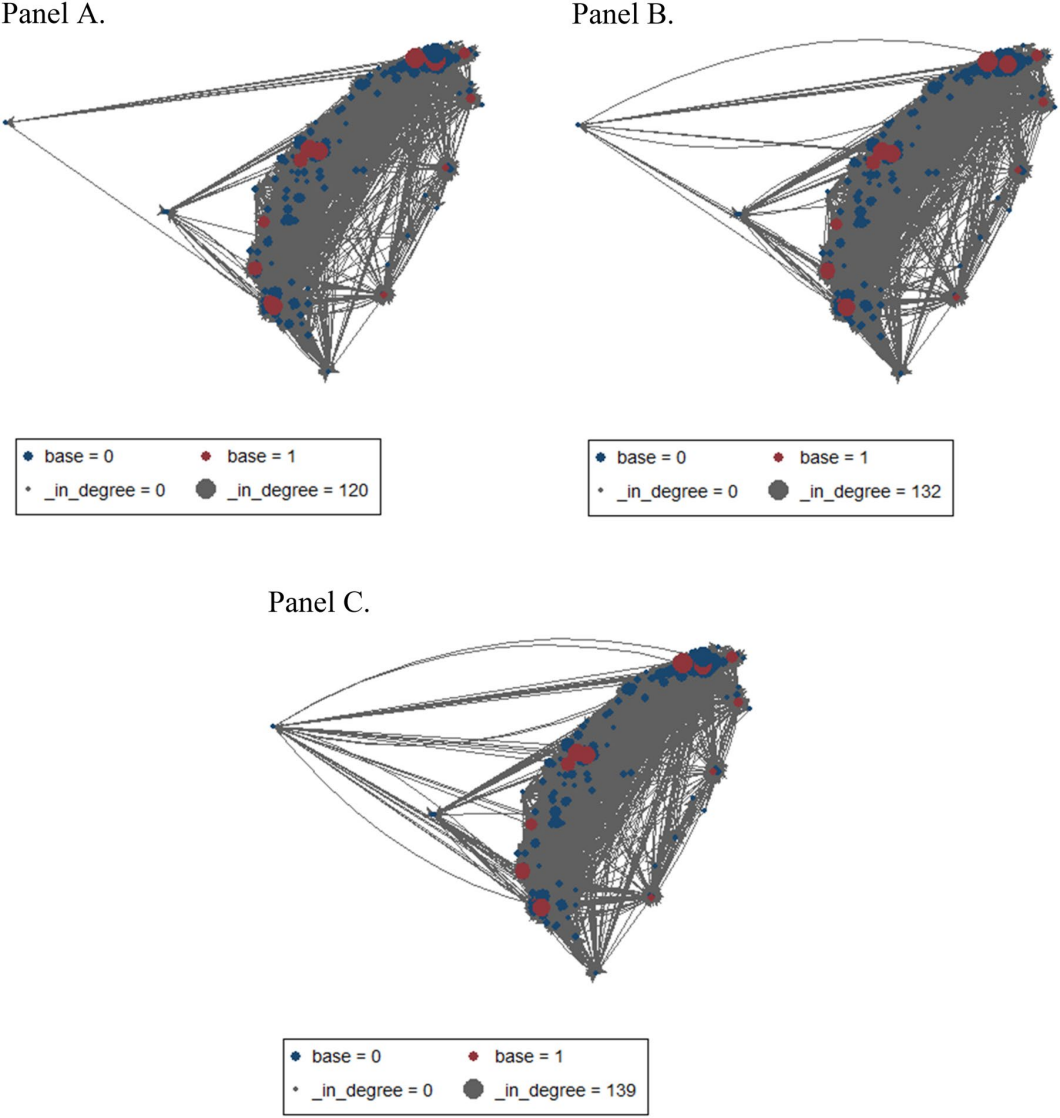
The dynamic changes in the network over time. Panels (**A**–**C**) represent the transfer network in 2014, 2015, and 2016, respectively. Red circles indicate base hospitals, and blue circles indicate non-base hospitals. The size of a node is proportional to its in-degree. Over the 3-year study period, the transfer ties increased over time. The figures were created using Stata 16.0 (stata.com).

Over the 3-year study period, the transfer ties increased over time (Fig. 5 and Online Supplementary video clip). This observation was supported by increased network density and in-degree centralization over time (Table 3).

**Table 3:** The change in network statistics over time, 2014–2016. There were increased network density and in-degree centralization over time.

	2014	2015	2016
Nodes (number of hospital-based EDs)	193	193	195
Ties (connections between hospital-based EDs)	5427	6138	6363
Density	0.15	0.17	0.17
In-degree centralization	0.48	0.52	0.55
Reciprocity	0.31	0.33	0.33

## Discussion

In this analysis of 218,760 emergency department (ED) transfers from a national electronic referral database in Taiwan, we found that the network structure largely aligned with the government-planned regionalized transfer network. We also identified key network metrics, at the global and ED levels, that can be used to track network performance and regionalization of care. This novel approach may be scalable and generalizable to other health care systems to identify inappropriate ED transfers and to evaluate the extent to which regionalized care is implemented.

In the United States, the national ED transfer rate was about 1–2%^27–29^, a figure that was comparable to ours (1%). A variety of factors have been reported to be associated with the decision to transfer: patient factors, insurance types, hospital factors, geography, resource level, and healthcare market^1,30,31^. In a previous study, the primary reason for transfers was for a higher level of care^29^, which was different from our primary reason of “requests from patients or families.” However, this may be a classification issue because the original question did not have a response choice of higher-level care. A qualitative study on transfer found that the reasons behind “requests to transfer” actually included the wish for a higher level of care^32^. Among the reasons to transfer, a lack of on-call specialists and hospital capability issues were still the main drivers for patient transfer in our study. Despite being transferred, about 6% of the transferred patients were discharged at the receiving hospital. In another study, 8% of the transferred patients were discharged after transfer^28^, potentially resulting in excess cost and burden to patients and families. Taken together, these numbers suggest that some transfers may be reduced, possibly through telemedicine or health information exchanges.

Regionalization of emergency care has been a subject of active research since the publication of the Institute of Medicine (IOM) report in 2006^2^. This landmark IOM report called for a regionalized, coordinated, and accountable emergency care system^33^. Regionalized care has been shown to have superior outcomes for several specific conditions^34^, and these are indeed among the most commonly transferred conditions in our study (e.g., stroke, myocardial infarction, and trauma). Our SNA further revealed a multiple hub-and-spoke, geographically regionalized network. All network statistics in our study also suggested a loosely connected (low density), moderately centralized system with localized clusters and key stakeholders (influential hubs with high influx). These statistics seemed to suggest a more coordinated and regionalized emergency care network instead of a fragmented system. The temporal analysis also confirmed that the network became more connected over time. Nonetheless, the size of Taiwan is only slightly larger than that of Maryland in the US. Coupled with a government-run, single-payer health care system, the administrative complexity is substantially reduced. Another metric suggestive of a more regionalized network is a lower number of transfer-out partners^28,31^. In our study, that number was a median of 5 partners over three years, compared to a median of 7 over a year from a previous US study^28^. Notably, the US study did not filter out less robust transfer partners as we did in this study, and therefore, the number of robust partners may be smaller.

Social network analysis also provides insights into the accountability of the emergency care system. For those regional hubs (base-hospital or specialized centers), do they receive transfers from too many partners (high in-degree) and too many patients beyond their capacity? The distribution of in-degrees suggested preferential attachment and a power-law phenomenon. The distribution of betweenness centrality also followed this scale-free pattern, indicating the importance of these hubs in the network. On the other hand, for smaller, resource-limited EDs, do they send patients to too many transfer partners (high out-degree), possibly deviating from an existing regionalized transfer protocol? The net connectivity plot also indicated negative traffic and possible financial losses for these resource-limited hospitals. From the outcome perspective, these extreme “distributor” EDs were also associated with a higher likelihood of patients being discharged in the receiving EDs, as shown in our study.

Using the network statistics as process measures, policymakers and administrators can identify outliers by performing an in-depth review of transfers, and facilitate load balancing during public health emergencies^35^. Besides process measures, visualizing network structure can discover structural issues, such as fragmentations, isolates, and divisions^12^, and targeted interventions can be implemented accordingly. In this study, the network analysis did not suggest a random or small-world network; rather, it suggested a more efficient hub-and-spoke network for transporting critically ill patients^36^. A recent study also mapped out this network pattern for stroke systems of care^37^. Moreover, our dynamic analysis of the network revealed increasingly dense and more transfers into the hubs, suggesting a favorable trend of regionalized care. Finally, coupled with patient outcomes, different networks can be compared, and best practices identified^38^. Alternatively, inappropriate transfers based on financial considerations^39^ or potentially avoidable transfers^40^ can also be identified for quality improvement purposes. As stressed in the IOM report, there is no “one-size-fits-all” solution to building the best emergency care systems^2^. Each region should strive to collect, link, and analyze transfer data in the aspects of structure, process, and outcome, thereby determining the best transfer model for regional healthcare providers, patients, and families.

This study has some potential limitations. First, receiving EDs were required to submit information within three days of receipt of patients. Thus, receiving EDs may have submitted information at different times during the three days, and some patient disposition may not be final (e.g., ongoing ED management). Second, we did not have information on the inpatient outcomes of the transferred patients. The next study would aim to associate different structural characteristics with a particular patient outcome for comparative purposes. Third, as with any large administrative data sets or patient registries, missing data or data entry errors may occur. The data collection form was online and structured, and we performed rigorous data cleaning, both of which may have mitigated this problem.

In summary, we used a novel SNA approach to examining the complex interhospital ED transfers nationwide. This approach helps unravel the transfer pattern between hospitals and visualizes if an existing network structure aligns with the planned regionalized transfer network. Understanding the network metrics helps measure network function over time, thereby tracking the structure and process aspects of regionalized care. Social network analysis can identify key players and patient flow in a network and has practical implications for regionalized care of time-sensitive conditions and beyond (e.g., disaster preparedness and outbreak containment).

## Supplementary Information

Supplementary Information 1.

Supplementary Video 1.

## Data Availability

The data that support the findings of this study are available from the corresponding author upon reasonable request.
