# Supplementary material for: Social network analysis of nationwide interhospital emergency department transfers in Taiwan
Source: Sci Rep. 2023 Feb 9;13:2311. doi: 10.1038/s41598-023-29554-4 (PMC9909649; doi:10.1038/s41598-023-29554-4)
Supplement: Supplementary file 1 — Supplementary Information 1. [file 41598_2023_29554_MOESM1_ESM.pdf]

Supplementary Table 1. Characteristics of the 14 base hospitals and population served in 2015.

Hospital name	Hospital level	ED categorization	ED annual visit volume	Number of hospital beds	Population size in the region
CGMH-KL	RH	Advanced	71,000	1,097	372,000
NTUH	AMC	Advanced	111,000	2,284	6,675,000
CGMH-LK	AMC	Advanced	169,000	3,404	7,112,000
VGH-TC	AMC	Advanced	68,000	1,587	3,326,000
CMUH	AMC	Advanced	161,000	1,764	3,272,000
CCH	AMC	Advanced	105,000	1,228	1,982,000
CGMH-CY	RH	Advanced	72,000	1,365	785,000
CMMC	AMC	Advanced	133,000	1,288	1,886,000
NCKUH	AMC	Advanced	103,000	1,193	1,886,000
VGH-KS	AMC	Advanced	88,000	1,482	2,779,000
CGMH-KS	AMC	Advanced	135,000	2,686	3,615,000
Po-Ai H	RH	Advanced	70,000	892	458,000
TZH	AMC	Advanced	52,000	987	331,000
MMH	RH	Advanced	43,000	465	221,000

ED = emergency department; RH = regional hospital; AMC = academic medical center.

Hospital abbreviations in the order of the network number: CGMH-KL=Chang Gung Memorial Hospital-Keelung; NTUH=National Taiwan University Hospital; CGMH-LK=Chang Gung Memorial Hospital-Linkou; VGH-TC=Veterans General Hospital-Taichung; CMUH=China Medical University Hospital; CCH=Changhua Christian Hospital; CGMH-CY=Chang Gung Memorial Hospital-Chiayi; CMMC=Chi-Mei Medical Center; NCKUH=National Cheng-Kung University Hospital; VGH-KS=Veterans General Hospital-Kaohsiung, CGMH-KS=Chang Gung Memorial Hospital-Kaohsiung, TZH=Tzu-Chi Hospital; MMH=MacKay Memorial Hospital.

Supplementary Figure 1. National Electronic Referral System, an online national electronic transfer platform.

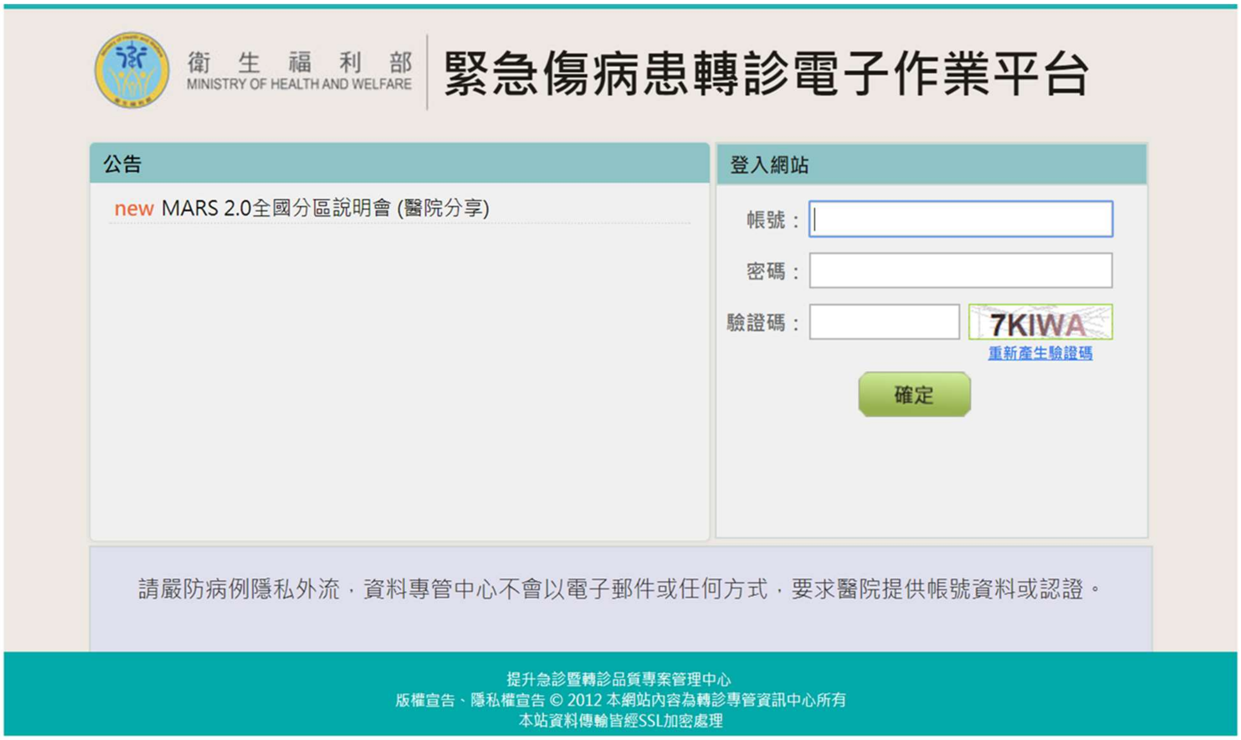

Supplementary Figure 2. The distribution of betweenness.

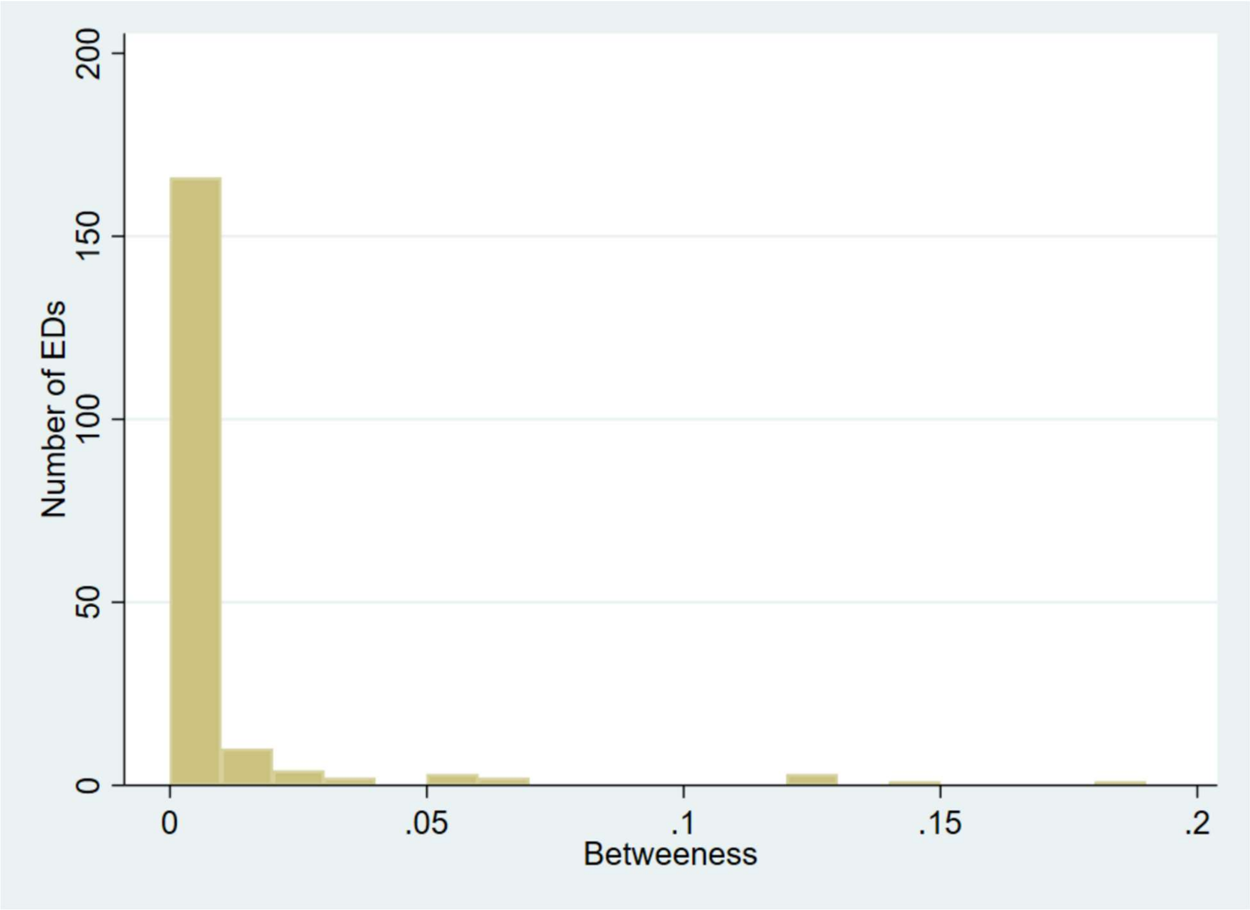

Supplementary Figure S3. The distribution of closeness.

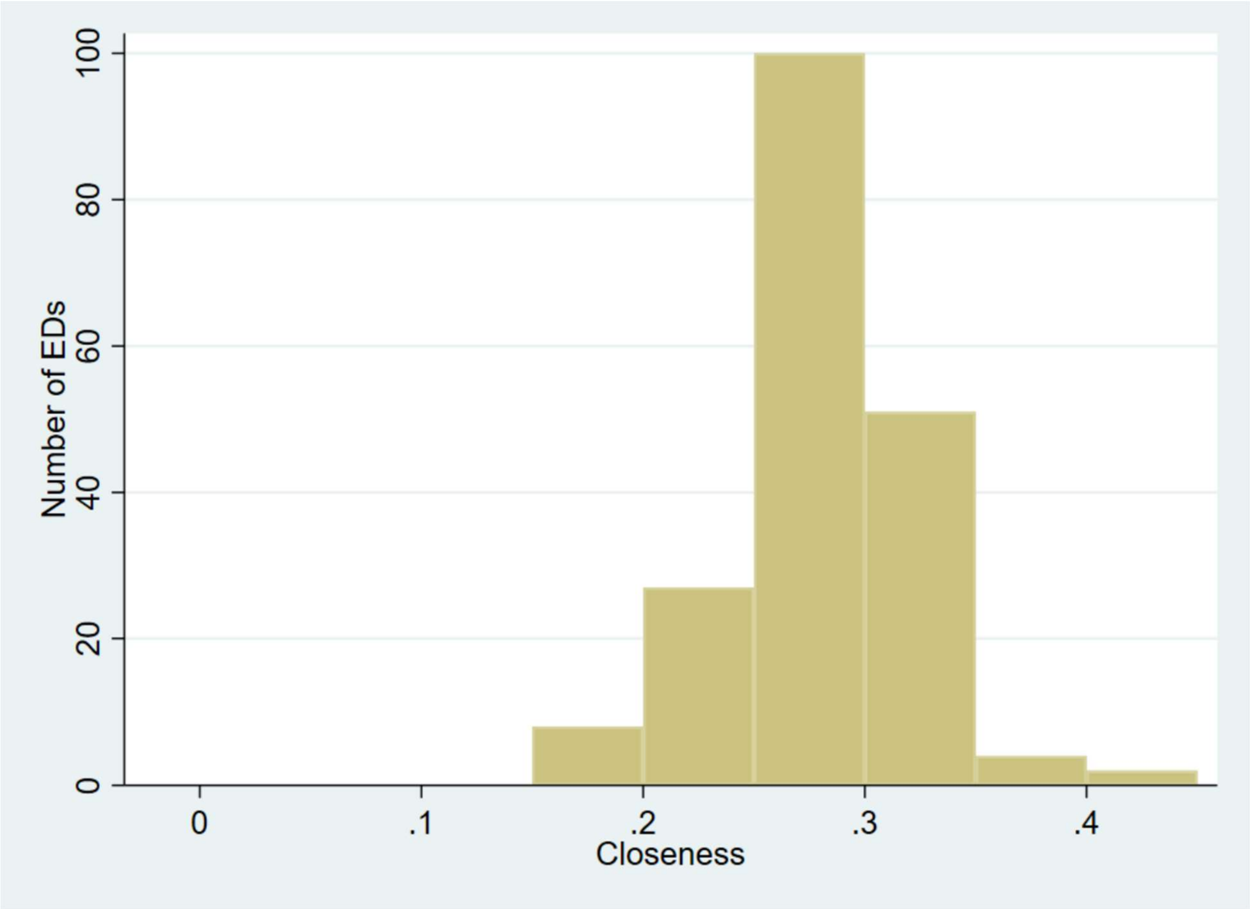

Supplementary Figure S4. The distribution of local clustering coefficient.

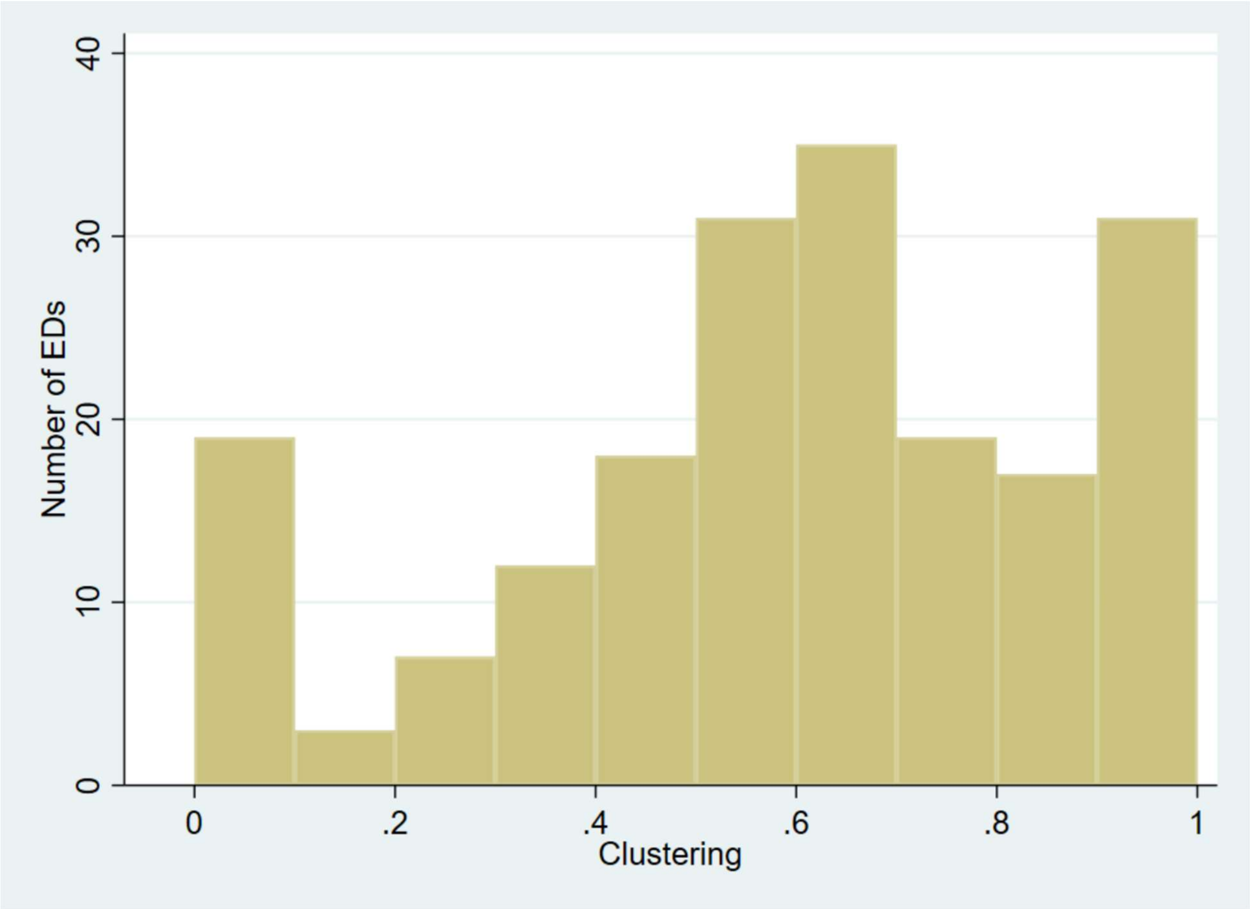

Supplementary Sample Code

```
*use a local network file
nwuse localfilename

*summarizing the network
nwsummarize,detail

*clustering coefficient
nwclustering

*in_degrees and out_degrees
nwdegree

*betweenness
nwbetween

*local components
nwcomponents

*dyads and triads
nwdyads
nwtriads

* mapping
nwplot, nodexy(longitude latitude) layout(nodexy)   edgesize (network,forcekeys (1 2 )) color
(base)
```

Supplementary Movie. A movie clip showing the network change over the three years.
